# Untangling approaches to management and leadership across systems of medical education

**DOI:** 10.1186/s12913-016-1391-9

**Published:** 2016-05-24

**Authors:** Kathy Hartley

**Affiliations:** Salford Business School, Lady Hale Building, University of Salford, Salford, M5 4WT UK

**Keywords:** Medical education, Management, Leadership, Competency frameworks

## Abstract

**Aims:**

How future doctors might be educated and trained in order to meet the population and system needs of countries is currently being debated. Incorporation of a broad range of capabilities, encompassed within categories of management and, increasingly, leadership, form part of this discussion. The purpose of this paper is to outline a framework by which countries’ progress in this area might be assessed and compared.

**Methods:**

Key databases and journals related to this area were reviewed. From relevant articles potential factors impacting on the incorporation of aspects of management and leadership within medical education and training were identified. These factors were tested via an online survey during 2013 with six members of a European Association of doctors who promote medical involvement in hospital management, including members from countries less represented in the health management literature.

**Results:**

A framework for analysing how management and leadership education is being approached within different systems of healthcare is developed and presented.

**Conclusions:**

More systematic work across a wider range of countries is needed if we are to have a better understanding of how countries within and beyond Europe are approaching and progressing the education of doctors in management and leadership.

## Background

Globally, discussions and reviews are underway as to how to align medical education with shifting socio-economic demands and health system needs. For instance, the World Federation for Medical Education ([1], p.10) stated that competencies (knowledge, skills, attitudes and behaviours) developed during postgraduate training should include “knowledge of public health and health policy issues and awareness and responsiveness to the larger context of the health care system, including e.g. the organisation of health care, partnership with health care providers and managers, practice of cost-effective health care, health economics and resource allocation [along with] ability to understand health care, and identify and carry out system-based improvement of care.” The Lancet Commission into medical education worldwide [2] has also highlighted a gap in systems-based and population-based education of all health professionals. On both sides of the Atlantic there is discussion about socio-economic change, based on the fact that, amongst other things, there is an ageing population who increasingly present with multiple, complex conditions, and a requirement for a more flexible medical workforce to respond to changing demands [3, 4].

Such proposals are, however, not going unchallenged. For example, in the UK the BMA (doctors’ representative body) has responded critically to the idea of a broader based training. Consultants (qualified specialists) have suggested that the reforms will be ‘dangerous for patients… [and carry a] risk of creating a cohort of senior doctors who are less well trained than the consultants of today” [5]. A particular concern has been that the proposed changes in the UK will result in medical graduates there openly competing for their first clinical post, immediately after undergraduate training, with graduates from medical schools in the European Economic Area and beyond. While on one hand this may appear a typically defensive stance on the part of UK doctors’ trade union, as Hodges and Segouin ([6], p.2] note, there is much discussion in European circles regarding the Bologna reforms, which apply to all higher education across Europe, and aim to align, in this case medical education “curricula, processes and outcomes so as to facilitate the movement of people and ideas across Europe.” As these authors note, there also appear to be quite different values between European educators and those in North America, as to what counts as quality in medical education, and subsequently how this is assessed.

Discussions on the future of medical education, and the need to incorporate training that enables doctors to contribute to managing, maintaining and reforming health service models of delivery, continues a debate that has been around since the introduction of new public management reforms [7]. Since the 1980s there has been a need for senior doctors to have management skills. Originally this was within formal, organisationally oriented roles such as medical or clinical director - variously referred to as professional-managers [7], hybrids [8, 9] and medical leaders [10]. Over the last decade the focus has turned to developing such management and leadership capability (increasingly conceptualised as medical or clinical leadership) within the younger generation of doctors to equip future hospital specialists who take up such roles, but also enable all doctors to contribute to the organisation of care from within their clinical role [11, 12]. This shift seems only likely to add to the complexity of education and training processes when one considers what is involved in managing and leading services, and that for early career doctors their future roles that may, or may not, include significant elements of management. As the paper will discuss, this raises questions about what they need to know and be able to do when.

Seeking to understand to what extent and in what ways systems of medical education are incorporating aspects of management and leadership is not meant to imply that such amendments are unambiguously a positive step. The existing literature, however, suggests that this is the emerging trend, albeit more prominent in some countries than others. Indeed, the purpose of doctors developing organisational capability, and the espoused benefits of their doing so do not go unchallenged. To some extent this is linked with issues regarding who is in the driving seat of change, and the ‘true’ nature of their motives. There are those in favour of addressing the historical neglect of management and leadership within medical education [10–12]. By contrast, there are those who question whether medical (and broader clinical) leadership can really deliver on policy expectations [13, 14], and whether incorporating such aspects into systems of medical education is simply a way of normalising managerial and organisation values and priorities which will ultimately prove to be at the expense of professional ones and detrimental to the profession and patients [15]. It has to be said that even those who strongly advocate medical leadership [16] note: “there are many subtleties and confusions of definition, meaning and terminology that need to be dealt with.” (p.174).

Whilst policymakers, the medical academy and professional associations are all important institutions in driving change, whether changes occur at the level of practice, as opposed to simply at regulatory and curricula level, may depend on local implementers [17]. In fact, when it comes to what is happening in practice, Kuhlmann and Von Knorring suggest that medical education has been “lagging behind” ([8], p.190) policy, even in places such as Sweden, which is relatively advanced in research terms with the doctors as managers/leaders issue. Other work suggests that in certain countries, such as Denmark, the profession has more readily accepted that doctors have a role in the organisation of health systems, and taken a more active role in educating doctors in aspects of management [18]. The idea that both policymakers and professional bodies impact on the extent and type of change occurring within systems of medical education, and that there is variability across countries is not new, and hardly surprising given the different ways in which more managed systems of care have been introduced across Europe. 

Notwithstanding the important debate about the precise purpose of doctors’ engagement with management and leadership, given the emerging trend and the fact that certain countries such as Australia Canada, the UK and US feature more prominently within the literature, in terms of amendments made to their educational curricula and provision of additional training in management and leadership for doctors, all of this raises certain questions. How far and in what ways are systems reshaping the structure and content of medical education in their countries, and how might we assess the extent of change? For instance there is an emerging body of work in Australia, but one that is less developed than in the UK and US [16]. Overall, the tendency is for country and initiative specific reports and, with limited exceptions [17, 18], there is little comparative work.

A lack of comparative work at international level is not unusual in relation to medical education [6, 19]. International comparisons include, for example, an open response survey of the introduction of longitudinal clerkships for medical students (a relatively recent advance in most countries) across 4 countries [20] and an examination of problem-based learning compared with a traditional teaching approach in five countries [21], with the latter being achieved through a collaborative international network of universities. Methodologically, comparison of educational approaches such as problem-based learning and traditional teaching methods is complex, due to variations in definition and conceptual problems regarding outcome variables. Research related to training has often struggled to get around issues of confounding, i.e. difficulty in understanding what particular inputs, or other factors, led to the outcomes seen [22]. Within country comparison of such things as the effect of clinical skills training across different institutions [23] has occurred, as well as within organisation comparison of new types of training intervention, such as simulation and computer based virtual patients, but often versus no training input, largely due to feasibility issues [24].

Whilst undoubtedly there are challenges to conducting comparative work, particularly across countries, scholars within medical education are calling for more work of this nature. Cook [24] for instance calls for work comparing different types of training interventions that is guided by theory and which identifies and controls for confounding variables. Hodges and Segouin ([6], p.3) argue that the Association for Medical Education in Europe is a highly active, well subscribed organisation (with participants from more than 70 countries) but that dialogue within Europe, and across the Atlantic, about the basics of medical competence might benefit from comparative work across countries and cultures. They suggest that existing international associations might be used to enable this (e.g. the World Federation for Medical Education) or new partnerships between national and regional education associations established.

In relation to medical management and leadership the existing literature provides some understanding regarding the type of changes that are currently occurring, and helps identify aspects that researchers might consider in future examinations. This paper moves on to review the existing literature, identify factors for comparison and propose a framework for examining change within systems of medical education. The framework is based around three main areas: how systems of education are structured, in terms of who (what parties) are involved in determining the undergraduate and postgraduate curricula and influences additional training provision; what is delivered and when during the formal medical training period, including mapping curricula against established leadership competency frameworks to highlight similarities and differences; what is delivered on an extra curricula and optional basis.

## Methodology

Firstly, established databases including Medline, EBSCO Source Premier, Emerald and Sage were consulted. Relevant literatures related to medical education and training and management and leadership development were reviewed. From relevant articles potential factors impacting on the incorporation of aspects of management and leadership within systems of medical education and training were identified. These were then explored via an online survey during 2013 with six members of a European Association of doctors that promotes medical involvement in hospital management, who were from countries generally less represented in the health management literature: Belgium, Denmark, France, Italy, Slovakia, Switzerland. 

The survey highlighted certain differences across systems in Europe, such as whether national or regional curricula exist and the range of parties involved in determining curricula. It also highlighted certain gaps in knowledge, for instance regarding precisely what aspects of management and leadership are formally included within curricula, or are addressed at individual institutions. This helped refine a framework for examining and comparing how different systems are approaching this issue. The paper now moves on to review the existing literature.

### Structures of medical education

The existing literature and survey carried out suggests that the overall structure of medical education is broadly similar across countries, generally being a period of undergraduate study of around 5 to 6 years, followed by a similar period of postgraduate study. The survey informants suggested subtle differences in terms of some countries having preliminary registration periods of registration prior to entry to postgraduate (specialist) training (e.g. France and Slovakia). The literature to date suggests both pros and cons with regards to whether aspects of management and leadership are incorporated earlier on within training, during the undergraduate or postgraduate stage or as part of continuing medical education (CME) [25, 26]. The issue of timing has perhaps become more salient with the emergence of ‘leadership’ as a shared activity and thereby a necessary behaviour of all doctors [11, 12, 28], as opposed to the earlier focus on senior doctors assuming formal roles with a particular ‘managerial’ aspect. Providing training before doctors become fully qualified hospital specialists may mean that there is a greater likelihood of them being effective more quickly once they take up a specialist post. However, they may also struggle to have much involvement until they are in such a position, by which time they may have forgotten much of what they learnt [26]. This of course might suggest that concepts and opportunities to lead need to be ‘drip fed’ throughout the duration of medical education.

### Parties involved in determining the curricula and the extent of standardisation

In some countries such as the UK a national level curriculum exists for each stage of training, whereas in others it is determined regionally, for instance in France and Switzerland. In fact the Swiss survey informant indicated that the undergraduate curricula there are developed at a regional level, whilst their postgraduate one is developed at a national level. In the Netherlands, as in Australia and countries across the Middle East, the Canadian model of medical education (CanMEDS) is influential [27] in determining the curricula. Yet there may be variances across the country via different universities and medical centres [17]. There is also variance in terms of the number of parties (individual universities, university associations, professional associations and colleges, national and regional policymakers) involved in determining curricula at each stage of education. Where there are more regional stakeholders involved this might, in part, explain why for instance specific frameworks for the development of leadership and management capability have not emerged.

### Incorporation of management and leadership

Traditionally systems of medical education have not incorporated managerial and organisational priorities [8], but over the last decade this has begun to change. For countries utilising the Canadian (CanMEDS) model of medical education [27] this is based around seven areas or roles in which doctors must become proficient to become ‘medical expert.’ One of these was previously ‘manager’ but in the 2015 version became ‘leader,’ to “more accurately reflect the scope of competencies that doctors are expected to demonstrate” ([29] p.2). Some suggest that the shift in discourse from manager to leader might offer doctors who have been reluctant to be managers a more attractive self-narrative [30], but it also reflects increasing complexity in health systems and associated calls for a wider constituency of doctors, across all levels, to contribute to service design and improvement from within their clinical role [11, 12]. This raises questions as to whether the knowledge, skills and attitudes needed by doctors have actually changes, and how they might best be addressed at each stage of doctors’ career.

Traditionally, management is associated with activities such as planning, devising ways to deliver products or services, achieve targets and monitoring for this, financial and budgetary responsibility, staff performance and marketing of services [31] – aspects incorporated to varying degrees, within clinical and medical director type roles [32, 33]. However, as Mintzberg [34] noted, one of the many roles of a manager is ‘leader,’ with doctors in such roles having being found to require and use leadership skills, in terms of championing new ideas, influencing, persuading and making sense of initiatives for their colleagues [33, 35]. Indeed, it is these relational skills in particular that doctors are now being encouraged to develop from a much earlier stage.

The recent revisions in the CanMEDS model challenge doctors to demonstrate ‘leadership’ in each of four key competencies: quality improvement, stewardship of resources, leadership in practice and management of one’s career. In other countries, such as the UK, a specific medical leadership competency framework exists (MLCF), constituting five areas of competency with this now formally incorporated into the undergraduate and all postgraduate curricula [35] and doctors expected to develop skills in these areas by the time they are fully qualified specialists. In Canada the Canadian Medical Association’s Physician Leadership Institute has a range of leadership programmes for doctors at early leader [36] practising leader and executive leader stage which map onto the broader Canadian leadership (LEADS) framework [37].

Broader professional frameworks also exist in Australia (Australian LEADS) [38] and the UK. In the UK development of a broader Clinical Leadership Competency Framework (CLCF) emerged from the MLCF [39] and has now been accepted by all the educational, regulatory and professional bodies. These broad clinical frameworks (i.e. not profession specific) frameworks in Australia, Canada and the UK are constituted of five skills areas, based around self-leadership, collaboration and leading others, change leadership and innovation, service management (outcomes related).

Following work that examined leadership patterns and behaviours within the UK NHS the NHS leadership Academy there has now developed a broader ‘Healthcare Leadership Model’ for staff involved in health and care work [40]. This consists of nine domains – ‘inspiring shared purpose’ (valuing service values, acting courageously and taking the initiative), ‘leading with care’ (supporting and caring for others in the healthcare team), ‘evaluating information’ (gathering data and feedback, spotting opportunities and risks, developing new ways to improve systems and processes), ‘connecting our service’ (collaborating across boundaries) ‘sharing the vision’ (communicating and inspiring), ‘engaging the team’ (style of leadership and valuing diversity), ‘holding to account’ (setting performance goals and standards, looking for ways to improve and innovate), ‘developing capability’ (looking at own and others skills, career strengths and plans) and ‘influencing for results’ (developing collaborative networks, engaging with a range of stakeholders and being persuasive). There are four possible levels of proficiency (basic, proficient, strong and exemplary) in relation to each domain.

Such recent developments suggest that ideas as to what constitutes leadership are continuing to evolve. While this may be understandable, doctors (and indeed other health professionals) might be forgiven for feeling uncertain as to precisely what it means to be a good medical leader, and by who and what standards they will be judged. Similarly, this creates issues for medical educationalists, some of whom have highlighted the lack of clarity surrounding how to develop doctors who are capable and willing managers and leaders [41].

While countries such as Australia, Canada and the UK may have similar competency frameworks, how far the behaviours and attitudes within these align with expectations of leaders and leadership internationally, and therefore might be useful in other countries, is debatable. Within the wider leadership literature the ‘Global Leadership and Organizational Behavioural Effectiveness (GLOBE) project, set up by House in the 1990s, has looked at behavioural characteristics of middle managers in sixty two cultures and found that of sixty-five leadership attributes which emerged, thirty five were culturally contingent [42]. The characteristics identified were narrowed down to six broad dimensions, three of which are viewed positively by most cultures, and reflect competencies within the aforementioned healthcare frameworks: the extent to which leaders are (i) charismatic or certainly values-based (able to inspire, motivate and expect high performance, based on firmly held values) (ii) team oriented (effective team builders, who develop a common purpose among members, and (ii) participative (involve others in decision making). Other behaviours, such as being ‘humane oriented’ (supportive, considerate, compassionate), ‘self-protective’ (ensuring the safety and status of individual or group members, which includes such things as face saving and being status conscious) or ‘autonomous’ (individualistic and independent) were viewed positively in some cultures e.g. South Asia, but more neutrally in others, such as the Nordic countries. It would therefore be interesting to examine what medical leadership means and how it is being addressed in a wider range of countries.

Where countries have developed leadership competency frameworks this might suggest that they are more advanced than others in terms of developing a wider range of doctors (and other professionals). However, it seems that this does not necessarily equate to doctors actually being involved at an organisational level. For instance, while the UK has developed a specific medical (and general) leadership framework, it has also been found to have less doctors in senior decision making positions (i.e. on hospital boards) than many other countries [43]. The existence of competency frameworks might then simply reflect a strong policy desire for the health professions to increase their engagement with the organisation of healthcare. Where the profession gets involved and starts to advocate medical leadership, as it has in the UK, via establishment of the Faculty of Medical Leadership and Management [44], this might indicate professional recognition of the need to engage, but also a desire to engage on its own terms.

Comparative research examining in the UK and Netherlands also suggests that the presence of such models such as CanMEDS and leadership competency frameworks is not necessarily an indication of what occurs in practice. Noordegraaf [17] found that change in the UK has so far been more related to teaching methods, with the content of teaching still largely focused on leading clinical teams, and little attention given to more organisational and managerial issues. Likewise, in the Netherlands most medical courses remained clinically oriented, despite recommendations from research institutes that a thorough training in management should be given during training. Earlier research has found that aspects of leadership and management are being incorporated into training, including administration and leadership in Norway and Finland, with health economics, leading in a political environment, the health system, organisational culture and change incorporated in Sweden [25]. The survey reported on here also noted that aspects relating to quality, safety and risk and health policy are covered in Belgium, France, Italy, Slovakia and Switzerland.

More recent views, including practitioners, do, however, support Noordegraaf’s conclusions. These argue that there is little coverage or opportunity to develop management and leadership skills [45–48] and highlight that some postgraduate (specialist) curricula are currently less fit for purpose than others in this regard [48]. In both the UK and Netherlands (and other countries) extra curricula training appears to be offering more substantial development in this area (as the paper will discuss shortly). Interestingly, much the same issues about access to training and development are highlighted in the US, where physicians have been involved in hospital management for a longer period [49]. Thus, even in countries that have management/leadership competency frameworks in place, and have supposedly amended their systems of education in line with these, implementation may hamper aims.

Lack of progress may, in part, be linked with broader issues over competency-based medical education, which has not been without its challenges. Medical educationalists have cited the need for faculty to accept changes to ways of educating and training doctors, and to develop the skills of those medical teachers who must train and evaluate students in the workplace [50]. They highlight a lack of uniformity in teaching [51] and the fact that competence is the result of individual and contextual dynamics, raising issues when it comes to assessing competency [52]. Early career doctors (those in specialist training) have also highlighted that shift working, frequent rotations and gaps in professional training globally which hamper their development of management and leadership skills [26, 46, 53]). Another reason suggested as to why they receive less development is that there is potentially a need for a quick financial return from investment in training and development. If this is the case, while prioritisation of existing leaders might be understandable given the current financial climate, it might mean that systems are in fact simply storing up problems for a later date, when early career doctors take up more senior roles. Overall, it seems that extra-curricula training, which doctors self-select to undertake, is available in a variety of formats, as the next section moves on to discuss.

### Training programmes versus education curricula

As a recent meta-analysis in the US [54], systematic review in Canada [55] and reviews in the UK [56, 57] have noted, there is currently a range of leadership development programmes in existence, with wide variations in approach and reporting of programme content and outcomes. One thing that stands out within the literature is the range of providers (universities, medical associations and unions, for-profit agencies, policy led academies, independent think tanks and so on). As such, programmes vary enormously in terms of pedagogical approach, content, duration, intensity, whether they are accredited or not (for instance by a university or professional body in Management and Leadership). For example, programmes range from individual workshops and 2–3 day programmes to more intensive interventions focused around experiential learning, leading live projects over 6–12 months, as well as MBAs (general and executive health MBAs) and Masters programmes [54–57].

It has been argued that training and development in healthcare should consider and learn from wider leadership theory and evidence [13]. More recent thinking here includes the idea that perceiving oneself as a leader and acting as such is an identity-centred process, one which occurs gradually through practice, social interaction, reflection and mastery [58, 59]. Indeed, Lord and Hall [59] argue that it is only as skills progress into the ‘expert’ domain that the identity and behaviour of leaders is guided by an understanding of the situation in which they operate and collaboration with others. How leader identities might be developed is an interesting area to consider within healthcare, give that doctors are heavily socialised, both formally and informally, as new recruits into a distinct set of values, norms and beliefs, known as medical professionalism.

Certainly there is a gradual trend in health systems towards providing opportunities to learn through live experience [54–57, 60, 61], which is in line with ideas of leadership as an identity-based phenomenon [58, 59, 61]. This is occurring through such things as leading specific quality improvement or change projects and working alongside managers at associated healthcare agencies. Noordegraaf et al. [62] reporting on programmes in The Netherlands where doctors have been involved in discussions and decision making regarding service improvement as part of their daily work suggest that initiatives that work *within* existing cultural frames of reference (styles, traditions and customs) may be more successful in terms of engaging the profession than those that do not. However, in a recent review of research into leadership development in general rover the last 25 years, Day et al ([63] caution that “while there is an assumption that experience plays an important role in developing effective leadership, research suggests that the empirical evidence for this assumption is far from definitive“(p.64). Another review has also noted that whilst action-learning opportunities are used fairly frequently across public sector institutions, including health, many fail to incorporate the necessary reflective element for real learning and change to occur [64].

### Evaluating the impact of education and training

Programme-specific evaluations suggest that, particularly the more intensive interventions, lead to doctors adopting a broader perspective on organisational issues and having enhanced confidence to lead both initiatives and colleagues [65–68] as well as developing important experience and skills in conflict management [68]. However, initiative specific evaluations have also highlighted certain problems, associated with the wider culture in which they occur. For instance, evidence suggests that new insights and skills may be undermined both by the cynicism of doctors’ own colleagues and ‘tokenistic’ support received from general managers [45, 65, 69]. More systematic reviews also suggest that many interventions are aimed at senior staff and lack a real focus on collaboration, even in countries where this is included in competency frameworks, and fail to incorporate a work based, experiential learning approach [55]. Overall, the longer-term impact of programmes is difficult to determine.

This, however, is not a peculiarity of the healthcare sector. The wider leadership literature highlights the complexity of establishing what works, in what group (e.g. what level of doctor and specialism) and why. Day et al highlight [63] that even where action and work based approaches do occur how we might capture ‘experience’ in terms of the knowledge, skills and practice emerging is complex, with the range of contextual factors involved in leader and leadership development also creating difficulties in establishing the causal chain empirically [68]. However, unlike private corporations, the healthcare sector funds professional education and training one way or another from the public purse, thus heightening the need to understand the impact of initiatives. In the UK the National Institute for Health Research had a call in 2014 for a systematic review of interventions to understand what works and why [70]) - a large scale, complex project and one requiring considerable investment. As the knowledge and skills needed by doctors is likely to vary to some degree across health systems, given the different ways in which they are structured, systematic reviews within and across countries might help to establish the type of interventions that are more successful in particular systems. Achieving this is likely to require the establishment of a range of indicators at a country level by which to assess outcomes versus objectives, both in the short and longer term*.* Clarity regarding the objectives and expected outcomes of training is also required [13].

In summary, certain countries such as Australia, Canada and the UK are relatively well represented in the literature on medical education and incorporation of management and leadership, having also developed competency frameworks which are broadly similar. The wider literature on leadership, however, suggests that while some leadership behaviours are universally supported, others are more culturally contingent. Thus, how far existing competency frameworks might prove to be of value across cultures is unclear.

Existing research also suggests that the existence of such frameworks does not necessarily mean that such countries have trained more of their doctors in management and leadership, or that doctors there are more involved in the management and organisation of health services. Other countries such as Denmark, Sweden and The Netherlands are also actively involved in training at least some of their doctors. Overall, it is difficult to untangle what education and training doctors are actually receiving in practice, even in countries where management and leadership is formally mandated, via incorporation within curricula, and where competency frameworks exist to guide this and other (optional) forms of training. Thus, making cross-country comparisons is particularly difficult. Looking at countries that are relatively advanced in this area, it appears that it takes time to determine and articulate precisely what skills and behaviours are required of doctors (and other health professionals).

Having considered the variations in the way that management and leadership is being addressed as part of doctors education and development the paper now moves on to outlining a framework that can be utilised to assess the existing literature within countries and collect data both within countries and from a comparative perspective.

### A framework for comparison

**Fig. 1:**
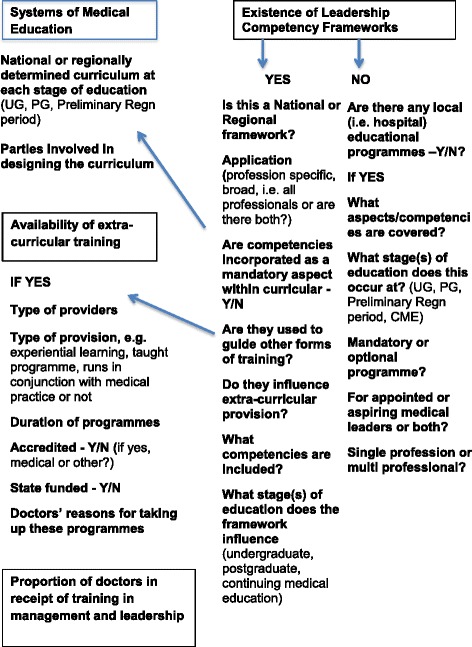
Framework for data collection and analysis

The above review highlights a number of specific factors on which systems of medical education across countries might be compared (see Fig. 1 for an overview).

Firstly, there is a need to consider how systems of education are structured, in terms of who (what parties) are involved in determining the undergraduate and postgraduate curricula and influence additional training provision. Where curricula are defined nationally, for example, there is the potential for greater standardisation within a country of management and leadership aspects within doctors’ training, and subsequently cross-country comparison. Identifying the parties involved (policymakers, universities, professional associations and so on) in determining curricula helps identify the source of potential change, such that shifts in policy and strategy can be tracked, as well as how these are implemented in practice.

Secondly, it is important to understand what is delivered and when during the formal medical training period, i.e. the content of curricula at each stage of training in relation to management and leadership. Where curricula are determined regionally or locally this will be quite an exercise in itself. Mapping content against existing leadership frameworks, even in rather different cultures, should help to determine how far systems are converging and in what areas there are differences across cultures*.* In addition, determining whether identified aspects of management and leadership included in curricula are offered on a mandatory or optional basis (e.g. offered as optional modules/pathways) and whether extra curricula training programmes exist is important, as it will provide insight into how seriously different systems are taking this issue.

Thirdly, research should consider what is delivered on an extra curricula and optional basis. This includes whether such management and leadership development programmes map against existing competency frameworks, who such programmes are aimed at (i.e. existing leaders, aspiring leaders, early career doctors or all levels of doctor), who they are run by (for instance professional associations, universities or private providers), the variety of types on offer (in terms of pedagogical and theoretical approach (model of leadership), duration and cost) and whether they are accredited are not. This will enable insight as to how far management and leadership is supported at a policy and professional level both within, and ultimately across, countries.

Fourthly, in relation to the above, gauging the proportion of doctors who have received education and training and desire it across a greater number of countries, through for instance surveys conducted via the academy or professional associations, will help establish how far systems have progressed in this area. Pre and post monitoring of changes to curricula (where this has not already occurred) would be useful. Where doctors are undertaking extra curricula programmes, undertaking qualitative work to establish why they are doing so (for example whether this is down to lack of formal input or dissatisfaction with the relevance of what is provided) and what might be done across different systems, should help policymakers and educationalists determine provision and provide cross- country learning.

Finally, where research identifies significant change to the curricula, or provision of additional training opportunities, some gauge of how much public money is being invested and particularly how return on investment, in the form of improvements in health service delivery, are being assessed, is an important consideration. The methodological implications of collecting this and the other data suggested will be discussed further in the following concluding section.

## Conclusions

Many countries are debating their needs regarding their future medical workforce, and how their systems of medical education might address the changes needed. In general, the need for broader clinical capability in conjunction with enhanced organisational capability, categorised increasingly as ‘leadership’ capability, is evident.

Mirroring the literature on medical education in general the existing literature regarding medical education and the incorporation of management and leadership is largely country and intervention specific, and suggests great variance even *within* national systems. Some countries have formulated competency frameworks to guide doctors’ education and training, but even where they have it seems that more intensive development opportunities are largely occurring outside of the formal system of education, via optional and extra curricula programmes, which vary enormously. To date, it is hard to gauge how far countries have progressed in terms of amending systems of medical education and/or providing additional training on such aspects and how similar (or not) changes are. Thus, despite healthy interaction at a European level, through such institutions as the European Association for Medical Education, opportunities for cross-national learning are presently limited.

The framework outlined here is one that can be utilised to assess the existing literature, determine gaps in knowledge and collect data both within and across countries to enable comparison. This will enable identification of variations in the way that leadership and management capability is being addressed across systems of medical education, such that interested parties such as the European Association for Medical Education may begin to have more meaningful debate. 

It should be noted, however, that the framework and research activities proposed here do present some methodological challenges, in terms of accessing a large number of institutions within any one country for a start. Where countries already have leadership frameworks in place to guide education and training, and are investing heavily in developing doctors, more systematic reviews as to what works, when and why are surely needed. Specific studies, commissioned by relevant bodies, are likely to be needed to ensure that confounding variables have been controlled for and that meaningful outcomes have been studied. More longitudinal studies are also required, and as other have noted, potentially more process oriented studies. Assessing the true benefit of all forms of training and development is complex, and requires clarity as to precisely what the objectives are at each stage of training. As has been noted, such clarity may well be lacking, and establishing this is a first point. A mix of quantitative and qualitative data, with mixed method approaches may be needed. For greater comparative work across countries there may need to be greater utilisation of existing international institutions and potentially new research collaborations created. If one considers, for instance, the GLOBE project this involved a substantial research team. As such investment in research will be needed.

In relation to education and training, assessing what happens, when, how, to whom and with what outcome is complex, challenging and resource intensive. The framework presented here provides a means of comparing across countries in a more systematic way. However, there are always likely to be some ambiguities researching in this area. Whether and how management and leadership development impacts on individual and organisational performance is akin to a search for the ‘holy grail,’-not only in healthcare, but it is particularly pertinent here, given that it is publicly funded, one way or another. Being able to assess outcomes versus input in a more robust way is likely to require the establishment of a range of indicators at a country level – as there is likely to be some cross national variation, due to different systems of healthcare and policy emphasis - by which outcomes versus objectives, both in the short and longer term*,* may be assessed*.*

While assessment of training inputs tends to be a rather ambiguous process, what is perhaps more certain is that the debate around the role of the doctor, their organisational as well as clinical responsibilities and how they might be educated to deliver what societies need in future, is set to continue.

### Declarations

This publication is supported by COST. This article has been published as part of *BMC Health Services Research* Volume 16 Supplement 2, 2016: Medicine and management in European public hospitals. The full contents of the supplement are available online at http://bmchealthservres.biomedcentral.com/articles/supplements/volume-16-supplement-2.
